# A cross-sectional study into medical students’ perceptions of healthcare regulation and self-reported compliance: a study conducted in the City of Al Ain, United Arab Emirates, 2016

**DOI:** 10.1186/s12909-018-1393-x

**Published:** 2018-12-13

**Authors:** Erik J. Koornneef, Paul B. M. Robben, Sandra Oude Wesselink

**Affiliations:** 1 Erasmus School of Health Policy & Management (ESHPM), Erasmus University Rotterdam, Rotterdam, The Netherlands; 2 Villa 42, Raha Gardens, PO Box 965118, Abu Dhabi, United Arab Emirates; 3 Medisch Centrum Twente, Enschede, The Netherlands

**Keywords:** Medical education, Perceptions of regulation, United Arab Emirates, Healthcare regulation

## Abstract

**Background:**

Although healthcare regulation is commonplace, there is limited evidence of its impact. Making sure that healthcare professionals comply with the regulatory requirements is a prerequisite to achieving effective regulation. Therefore, investigating factors that influence compliance may provide better insights into how regulators can be more effective. This study aimed to find out if medical students’ perceptions of regulation in the United Arab Emirates are associated with self-reported regulatory compliance.

**Methods:**

In the cross-sectional study, we administered a structured questionnaire to students of medicine with different statements concerning their perceptions of healthcare regulation and self-reported compliance. The statements included statement regarding the legitimacy, fairness and regulatory performance, as well as the risk to getting caught and being punished. The association between perceptions and self-reported compliance was analyzed using multiple regression models.

**Results:**

One hundred and six Year 3 and 4 pre-clinical medicine students (56.4% response rate) completed the survey. Almost 40% of the students rated their level of awareness and understanding of regulation as Good or Very Good., despite their lack of direct contact with the regulatory authorities (less than 10% reported monthly or more frequent contact). Self-reported compliance was high with almost 85% of the students either agreeing or strongly agreeing with the four compliance statements (mean score 4.1 out of 5). The findings suggest that positive perceptions of the regulator’s performance (β 0.27; 95% CI 0.13–0.41), fairness of the regulatory processes (β 0.25; 95% CI 0.11–0.38) and its legitimacy (β 0.23; 95% CI 0.05–0.41), are stronger associated with compliance than the perceived risks of getting caught and being punished (β 0.10; 95% CI -0.04 – 0.23).

**Conclusions:**

To improve compliant behavior, healthcare regulators should pay more attention to their own perceived performance, as well as the perceived fairness and legitimacy of their regulatory processes rather than focusing on more traditional methods of deterrence, such as perceived risk of getting caught and being published.

**Electronic supplementary material:**

The online version of this article (10.1186/s12909-018-1393-x) contains supplementary material, which is available to authorized users.

## Background

One of the central tenets within the study of public service delivery is the notion that public services should deliver the greatest benefit to the maximum number of people [1]. Regulation plays an important role in this as it aims to oversee the quality and performance of services [2]. In the healthcare context, regulation consists of mandatory requirements, such as standards, laws or directives and tends to focus on basic safety elements to protect public health [3] and improve quality of care [4]. The assumption is that a positive effect will be realized if these regulatory requirements are complied with in full [4].

However, researchers have reported a lack of empirical evidence regarding the effects of regulatory interventions on the level of compliance as well as the actual quality of healthcare and patient outcomes [4–6]. A study undertaken by the RAND Cooperation into the regulatory mechanisms of six countries concluded that the overall evidence of the effectiveness of regulatory strategies towards ensuring care quality and safety at system level is still scarce [7]. One of the biggest challenges in this context is the healthcare professionals’ lack of compliance with requirements which contributes to a poor quality of care and put patients at risk [8]. Even a simple requirements such as appropriate hand hygiene is known to be one of the most effective ways of improving patient safety [9] and it is widely endorsed by regulators as a mandatory requirement [10]. Despite these efforts, actual compliant behavior is lower than the recommended guidelines, around 40% [11].

Regulation involves rules that must be followed but in the healthcare context very few empirical have looked at why some organizations or individuals display compliant behavior and others do not [4, 6]. This study will take a closer look at the reasons why some people comply with regulatory requirements and others do not by focusing on the role of perceptions of procedural justice and deterrence.

The traditional viewpoint of compliance with regulation has primarily concentrated on deterrence: people are thought to obey rules and laws because there are penalties and incentives [2]. From this point of view, people are “amoral calculators”, interested and motivated by their self-interest. This view supports the notion of a regulatory approached characterized by the strict application of formal enforcement mechanisms [12]. However, studies across different settings have found that deterrence with penalties and rewards has a small influence on people’s compliance behaviour [13] and sometimes even the opposite effect [14].

In contrast, several studies have found that a regulatory process that is procedurally fair is an important motivating factor for compliance in different areas, such as residential homebuilders’ compliance with regulations [15], business firms’ compliance with environmental protection regulation [16], taxpayers’ compliance with taxation rules [17] and even patients’ adherence to doctors’ medical recommendations [18]. As Healy [6] puts it “the evidence is that most people and most organizations respond well to a respectful and supportive approach”.

In his seminal work on compliance and regulation in the 1980s, Tom R. Tyler studied people’s self-reported compliance with the law. In the so-called Chicago study, Tyler [19] looked at what factors shape compliance and what make people obey laws and regulatory requirements. One of his main findings was that when people are treated fairly by authorities, they are more likely to comply with requirements, because there is a relational bond. This is also known as the procedural justice model which leads to legitimacy, the belief that rules and regulations should be obeyed by virtue of who made the decision or how the decision was made [20]. The perceived fairness of the procedures and processes involved in regulatory decision making, as well as the perceived treatment one receives, are known to be important factors influencing compliance [17]. There is growing empirical evidence that this regulatory approach focused on cooperation has a stronger impact than the more traditional, deterrence based approach [21]. This emphasis on legitimacy also influenced Ayers and Braithwaite [22] to propose the theory of ‘responsive regulation’ that focuses on regulation based on trust and asserts that regulator should be flexible and decide to utilize a range of regulatory measures and strategies depending on what is required. These regulatory measures and strategies can range from persuasion all the way to legal penalties.

**Fig. 1:**
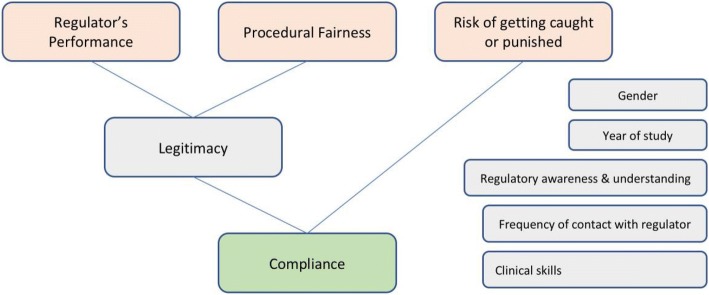
Conceptual model (adapted from Sunshine and Tyler [23]))

The Figure below (based on Sunshine and Tyler’s original model [23]) explains the predictive model for compliance in a conceptual manner. We propose two antecedent conditions of legitimacy: the regulator’s performance and the perceived procedural fairness [24]. Legitimacy itself, together with the perceived risk of getting caught and punished are considered to be the strongest antecedents to the self-reported compliance (Fig. 1).

The study took place in one of the main medical and health sciences university of the United Arab Emirates (UAE). The UAE is a federal union of seven states (Emirates), established in 1971. The country has seen a huge economic and population growth, from an estimate of 287,000 inhabitants in 1971 to around 9.1 million population in 2017 [25]. The UAE consists of a large portion of expatriates workers (around 88.5%) and a small number of UAE Nationals (around 11.5%) [26, 27]. In terms of healthcare regulation, the UAE is quite fragmented [28] and the two largest emirates, Dubai and Abu Dhabi, have their own regulatory authorities that are responsible to provide oversight and control over the facilities and professionals in their respective jurisdictions.

At Federal level the Ministry of Health is responsible for regulating the activities of the remaining facilities and professionals [29]. The UAE has a relatively well performing healthcare systems in the region, for example, Legatum Prosperity group ranked the UAE 28th out of 149 countries [30] and it has made significant progress in establishing major academic and research institutions [31].

The hypothesis of this study is that a more favourable perception of regulation in terms of legitimacy is associated with higher levels of self-reported compliance with regulatory requirements in the healthcare context. The objective of this study was to explore and investigate medical students’ perceptions of the healthcare regulatory environment. This was carried out by assessing the perceptions of medical students across a range of legitimacy related constructs such as procedural fairness, performance, risk and empowerment and the self-reported levels of compliance.

## Methods

### Study design

To test the association between legitimacy and other factors and self-reported compliance, a cross-sectional survey was designed to elicit the views and perceptions of the participants. All students in the medical school were invited to participate in the study. The research proposal received approval from the relevant Research and Ethics Committee in January 2016 and the study was carried out over a two-day period in April 2016.

The survey instrument focused on the general views and perception of regulation in healthcare rather than specific personal experiences. The survey instrument was developed in consultation with the university’s Faculty of Medicine and it was prepared after a thorough review of the medical literature, identifying distinct items that have been used in other studies [17, 18, 32, 33] to measure the relevant dependent and independent variables.

### Study population

The country’s relevant educational authority has accredited the university to provide the medical education program [34]. The faculty offered a six-year Doctor of Medicine, M.D. Program to UAE Nationals. The medical faculty ranked amongst the best medical schools in the GCC region and the university took in around 100 new medical students annually in 2016/2017. At the time of this study the university served as the primary source of medical education for citizens of the UAE [35]. The first two years of the six-year curriculum included a clinical foundation module that provided students with basic knowledge of the principles underlying clinical practice. Even though medical education in the UAE has received national accreditation, the undergraduate program has been characterized as being too focused on classroom based education, rather than hands on training [36].

As part of the university’s first year curriculum for medical students, the university offered a short, general orientation into the health care service provision in the UAE, including the regulatory role and function of the relevant authorities. Despite this it was assumed that students had a limited experience and understanding of the regulatory context and the survey briefly described the role of the regulatory authorities in healthcare, with a clear short description of the main regulatory functions.

### Data collection

All third, fourth, fifth and sixth year medical students (333 students in total) received an invitation by email from the Assistant Dean of the Medical Faculty to participate in the research study. However, the students were required to visit the Research Lab in person, as the research study formed part of a wider study into regulatory compliance. This meant that final year students (fifth and sixth year, 145 students) were unable to take part as they were enrolled in residency programs in various hospitals and clinics across the UAE. The total (third and fourth year) student population was therefore 188. Upon registration, each participant received a unique identifier and each student was asked to complete the Consent form. Once consent was granted, the participants were brought to a classroom by a Research Assistant where the students could complete the survey.

### Study variables

The survey consisted of two sections – the first section dealt with measuring the students’ views and opinions regarding regulation as well as their self-reported compliance and the second section asked general, background questions about the students’ experience as well as their self-reported compliance rating. In the first section students were asked to indicate their level of agreement on a five point Likert scale with eighteen statements. The scales ranged from one (Strongly Disagree) to five (Strongly Agree). The survey items assess the medical students’ appraisal of the healthcare regulatory authority in the UAE across the main facets of legitimacy: perceived risk of detection, performance and empowerment of regulatory authority and fairness. The survey also contained numerous questions about students’ self-reported awareness and understanding of regulatory requirements.

In our study the dependent variable, compliance with regulatory requirements, is measured by the medical students’ self-reported compliance. The independent variables, related to the students’ perceptions, are measured using statements describing statements relating to legitimacy, fairness, risk (the perceived likelihood of being caught and punished for not complying with the regulatory requirements) and the regulatory authority’s performance or empowerment (views regarding the authority and power of the regulatory authorities). In addition to this, the students’ prior knowledge, understanding and experiences with regulatory authorities was measured. In this study, we explored medical students’ perceptions of four independent variables (perceptions of the regulator’s legitimacy, fairness, performance and estimates of risks) and one dependent variable (self-reported compliance). The different statements (see Additional file 1) were derived from other studies into the relationship between legitimacy and compliance in the fields of compliance with taxation, justice and policing [17, 23, 24].

### Compliance

Four questions were devised to assess the dependent variable, self-reported compliance (Cronbach’s Alpha: .393). These items included statements such as “My friends and family would describe me as somebody who complies with rules and regulations”, “I try very hard to follow relevant guidelines and requirements from regulatory authorities” and “In general, I tend to comply with what is expected of me by regulatory authorities”.

### Legitimacy

Legitimacy is defined as the property of an authority or institution that leads people to feel that authority or institution is entitled to be deferred to and obeyed [32]. Put simply, legitimacy is the perception that one “ought to obey” another. The independent variable related to the theory that people are more inclined and willing to follow rules and regulations if they believe these are legitimate, i.e. the regulations are desirable, proper and appropriate in line with societal norms, values and beliefs [37].

This study measured legitimacy as the perceived obligation to obey and trust in regulatory authorities, with five items (Cronbach’s Alpha: .475), such as “You should accept the decisions made by the regulatory authority, even if you think they are wrong” and “The laws and regulation issued by the regulator are consistent (in line with) the views of residents in the UAE”.

### Fairness

The survey instrument contained four items relating to the fairness of the decision-making and treatment (Cronbach’s Alpha: .799) such as “The regulatory authorities in the healthcare field make their decisions based on facts, not opinions” and “Regulatory requirements are applied to all people consistently”. The two key dimensions of procedural fairness judgments are fairness of decision making (voice, neutrality) and fairness of interpersonal treatment (trust, respect) [19].

### Performance and empowerment

The students’ perceptions of the performance of the regulatory agencies was measured by asking how effective they perceive regulatory authority is and the effects of the regulatory actions. Two items (Cronbach’s Alpha: .635) were included: “Regulations such as standards, directives and policies are needed because they have a strong, positive impact on the quality of care delivery” and “In my opinion, the regulatory authorities are effective in improving the quality of health care delivery”. The students were also asked to what extent they agreed that the regulatory authority should be autonomous and have power to make decisions: “The regulatory authority should have the power to decide which regulatory requirements are the most important”.

### Risk of getting caught or punished

The survey included two items (Cronbach’s Alpha: .303) that looked at the students’ perceptions regarding the likelihood of being caught and punished for not complying with regulatory requirements, including “It is likely that you get caught and penalized if you break any rule or regulation”.

### Statistical analysis

The students’ responses were coded and the data was analyzed using SPSS (v22, IBM Inc.) software.

In order to analyze the relationship between the independent and dependent variables, the scores were calculated for each item by allocating a weight between 1 and 5, with a weight of 1 for “Strongly Disagree” and 5 for “Strongly Agree”. The scores for each item were added up and divided by the total number of completed items. Missing data were excluded from the calculation. In total 106 surveys were completed and each survey included 23 items (see Additional file 1). Seven surveys were incomplete with no more than one item not filled in. The average score for each variable was calculated by adding up the average score for the relevant items and then dividing this score by the number of items for the variable.

In order to test what factors influenced self-reported compliance, we performed an ordinary least squares regression analysis using the indexes of legitimacy, risk, performance evaluation, procedural fairness, awareness and understanding, as well as the frequency of contact, self-assessed clinical skills evaluation and demographic variables. From the regression model’s beta and 95% confidence intervals were derived. *P*-values of < 0.05 were considered to be significant.

## Results

A total of 106 students agreed to participate in the study (response rate 56.4%, 106/188), 83 participants were female (78.3%) and 23 were male (27.1%), see Table 1 below. All participants were UAE nationals, 23% male and 77% female.

**Table 1:** Participation rates amongst male and female students

Year	Male		Female	
	No. participated	Response pate	No. participated	Response pate
3	18	75%	37	47%
4	5	25%	46	70%
Total	23	52%	83	58%

In terms of the frequency of contact with the regulatory authority, a high percentage of students (62.3%) had never dealt directly with a regulatory authority, whilst 27.4% had infrequently dealings with the regulators, see Table 2 below.

**Table 2:** Frequency of contact with the regulatory authorities (*n* = 106)

	Never	Infrequent	Monthly	Weekly	Daily
In the past 12 months, how often you have been in direct contact with regulatory authorities such as HAAD, DHA or the UAE Ministry of Health?	62.3%	27.4%	8.5%	1.9%	0.0%

The respondents were also asked a number of background questions. Overall, the majority of students rated their own clinical skills and competencies as “good” (55.7%) or “very good” (9.4%), see Table 3 below. Furthermore, over 60% of respondents indicated that they had an average or above average understanding and awareness of the regulatory requirements.

**Table 3:** Clinical skills and awareness/understanding of regulatory requirements (n = 106)

	Very Good	Good	Average	Poor	Very Poor
Overall, how would you rate your awareness and understanding of the current regulatory requirements in the UAE?	8.5%	31.1%	21.7%	17.9%	20.8%
I would rate my own clinical skills and competencies as	9.4%	55.7%	26.4%	6.6%	1.9%

The highest mean on the four independent variables was the performance and empowerment of the regulatory authority: 4.1 out of 5. The legitimacy variable had the lowest mean score, 3.3, followed by perceived fairness (mean: 3.8) and the perceived likelihood of being caught and penalized for breaking a rule or regulation (mean: 3.8). In order to measure the dependent variable, self-reported compliance with regulatory requirements had a mean score of 4.1 out of 5.

**Fig. 2:**
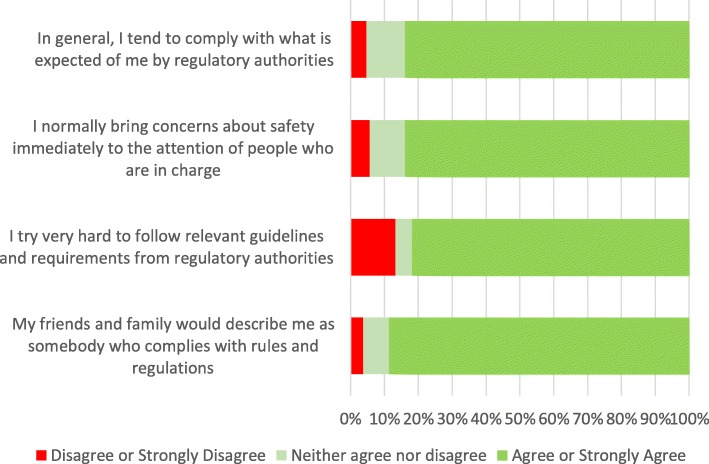
Self-reported compliance amongst medical students in the UAE (*n* = 106)

An average of almost 85% of all respondents either agreed or strongly agreed with the four compliance statements, see Fig. 2 below.

Finally, this analysis enables us to estimate the strength of the relationship between each independent variable and the dependent variable. The results of our analysis are shown in Table 4 below.

**Table 4:** Examining variables associated with self-reported compliance

	β	95% Confidence Interval
Legitimacy	0.23*	0.05–0.41
Fairness	0.25**	0.11 – 0.38
Performance & Empowerment	0.27**	0.13–0.41
Risk	0.10	-0.04 – 0.23
Contact with regulator	0.09	−0.05 – 0.23
Regulatory awareness and understanding	0.04	−0.04 – 0.12
Clinical skills and competencies rating	−0.05	−0.18 – 0.07

*p < .05

**p < .001

The strongest relationship was between legitimacy and compliance (β 0.23; 95% CI 0.05–0.41), fairness and compliance (β 0.25; 95% CI 0.11–0.38) and regulatory performance and compliance (β 0.27; 95% CI 0.13–0.41).

## Discussion

Considering that one of the core objectives of regulation is to oversee or control activities that are socially valued [38], it is important to find out more about how the people who are the subject to the regulatory requirement perceive the regulations. As noted, there is growing empirical evidence that a positive perception of the regulatory authorities’ fairness, performance and legitimacy increases the likelihood of compliance in fields such as law and order and taxation [24]. This procedural justice model of compliance has remained almost entirely based on research evidence from the United States [39] and has only been used in a small number of areas [40]. Using the extensive body of evidence [19], this is the first ever study conducted exploring the relationship between the perceptions of regulation and self-reported compliance amongst medical students.

We would like to make three general observations about the results before we look at the extent to which deterrence and procedural justice have an influence on compliance with regulatory requirements.

First of all, in terms of the UAE’s regulatory context, researchers [28] have commented on the consequences of a fragmented regulatory system leading to confusion and complicated rules governing each Emirate. However, over 60% of all respondents rated their awareness and understanding of current regulatory requirement as average or above average. This is even more remarkable considering the high number of students (more than 90%) that had limited or no contact with the regulatory authorities. It is also noteworthy that the majority of medical students rated their own clinical skills and competencies highly (more than 66% of students rating their skills and competencies as very good or good, see also Fig. 1), considering that other studies observed that in the UAE “undergraduate medical education continues to be comprised of long hours in the classroom and frequent written examinations, but limited hands-on training” [36]. Other studies have found similarly high self-reported skills rating [41, 42], with a negative relationship between years of experience and self-assessment ratings of clinical skills and competencies. One possible explanation could be that the lack of experience has impacted the overestimation of their own skills and competencies as well as the compliance levels.

**Fig. 3:**
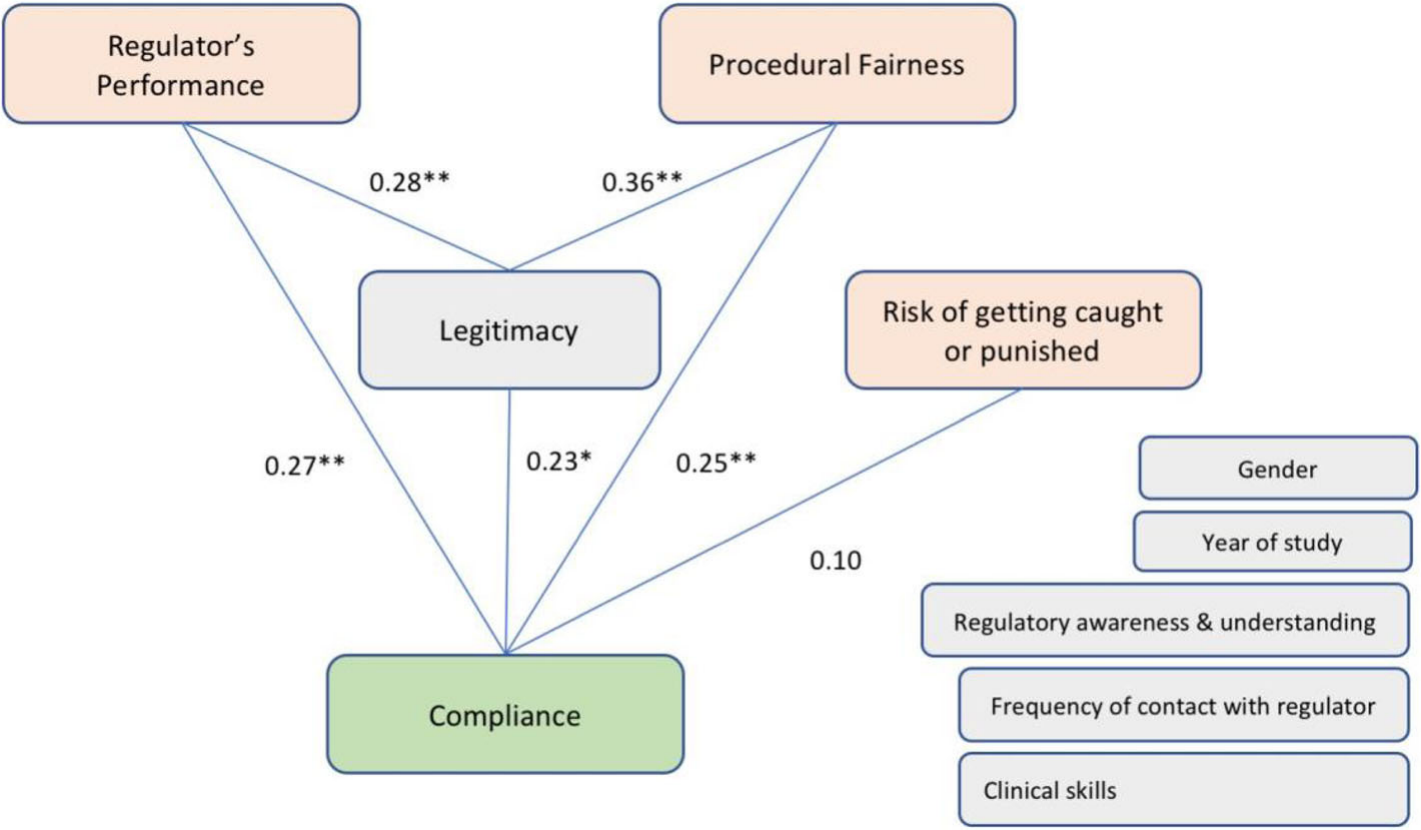
Our findings, using the conceptual model (adapted from Sunshine and Tyler [23]*)*

Finally, another interesting observation is the participants’ high average compliance score. For example, almost 90% either agreed or strongly agreed with the statement “My friends and family would describe me as somebody who complies with rules and regulations”. In contrast, several researchers have found suboptimal levels of compliance in similar settings in the UAE such as adverse drug reporting [43], over the counter sales of antibiotics [44] and adherence to diabetes medication [45]. Since these are self-reported ratings, it may not necessarily translate into actual compliant behavior.

These three observation are interesting from al regulator’s point of view, as it indicates the high level of support for healthcare regulation, as well as high scores on self-reported competencies and compliance. A team of researchers who evaluated the regulatory system for healthcare professionals concluded that the UAE had made significant progress in developing and implementing best regulatory practice [27]. Our study supports this view insofar that medical students had a largely positive view of the performance of the regulatory authorities in the UAE, with almost 86% of all students agreeing with the statement that regulatory authorities in the UAE are effective in improving the quality of health care delivery. A recent study exploring UAE medical students’ perceptions of international accreditation for medical education found a similarly high level of support [34] for this particular type of regulatory intervention.

In terms of the factors influencing compliance, the results described in the previous section support our hypothesis that procedural justice related variables have a stronger effect on compliance than deterrence as measured by the perceived likelihood of getting caught and being penalized.

As Fig. 3 below indicates, both regulatory performance and fairness are also associated with legitimacy, a finding similar to other studies [17, 32, 46]. The other variables, such as gender, clinical skills, regulatory awareness and understanding, etc. do not have a significant association with the compliance ratings and there are no discernible trends between these variables and the self-reported compliance.

Similar to other studies [23], procedural fairness was the primary driver of perceptions of legitimacy (beta = 0.36). The perceived likelihood to get caught or be punished (beta = 0.10) does not have a significant association with compliance rates. These findings are consistent with studies in other fields, such as policing [47] and law. [17]

In terms of measuring this relationship in a healthcare context, our research has found similar results as two other studies. The first study [18] found strong support for the argument that when healthcare authorities use fair procedures, patients are more likely to accept their recommendations. The second study [33, 48] concluded that the satisfaction of nursing home owners is more strongly associated with the fairness of the inspection process than the actual favourableness of the regulatory outcomes.

Obviously, it should be noted that the largely positive attitudes towards regulation as well as the high levels of self-reported compliance may not necessarily be sustained over time and result in positive behaviors and attitudes of physicians in the future. It is encouraging to note the positive attitude and intention to comply amongst current students. Other researchers [49] have found that healthcare professionals’ intention to comply appears to have a reasonably strong relationship with actual compliance. In terms of medical education, more attention could be given to ensuring that medical students are empowered to comply with regulatory requirements and meet the healthcare needs of the society.

### Limitations of study

The overall response rate was reasonably high (56.4%) and a number of students who had intended to participate contacted the medical school beforehand to explain that they were unable to attend in person due to other commitments. The response rate may have been higher if an additional, online survey option had also been made available to the students. The medical school is the primary source of medical education in the UAE [35] and each year around 80–100 students graduate from this particular school and only around 130 students apply for residency programs in the UAE every year [34]. Therefore it could be argued that participants are reasonably representative of the slightly larger population of medical students. The sample did not differ from the total Year 3 and 4 population in terms of gender (sample: 78% female vs. 77% female for the total population).

The study assessed the self-reported rather than the observed compliance levels. However, self-reported compliance in the healthcare field is not always associated with actual compliance [50]. In other words, a high level of self-reported compliance may not translate into a high level of actual, observed compliance making it difficult to draw any major conclusions from surveys based on self-reported compliance levels.

Another limitation of this study is the lack of students’ exposure to regulatory authorities, over 60% indicated that they never had any contact with the regulatory organizations. Since the students were only in their third and fourth year we would not have expected them to be overly engaged with the regulatory authorities as their professional licensing process would only commence after graduation. At the same time, students did indicate a high level of awareness and understanding of the regulatory requirements, perhaps as a result of their pre-clinical, practice based training, involving learning courses focused on real life examples.

Since the medical students were relatively unfamiliar with the regulatory requirements, they may have tended to provide responses which they deemed to be socially desirable. The high, self-assessed scores on awareness, clinical skills and compliance may be an indication of a high level of social desirability [51]. Other studies in the UAE with similar self-assessment methods found equally high rating in terms of competency [42]. These high scores may indicate that the medical students responded in a socially desirable way and some of the study results should be interpreted with caution.

Finally, the medical students had limited clinical experience and exposure to regulations or regulatory authorities. This may have resulted in a overestimation of the importance and impact of regulation.

## Conclusion

Regulation based on trust and fairness is often more effective than more traditional, rational choice approach [33], with a focus on deterrence. This study aimed to contribute to the growing body of knowledge [5, 52] into the role of procedural justice and its effects in healthcare. As we have seen in this study, negative motivations arising from a fear of the consequences of violating regulatory requirements is not as strong a factor when it comes to influencing compliance compared to positive or affirmative motivations such as creating a sense of trust in the regulatory authority’s work and the obligation to comply [53].

Considering that a lack of compliance with regulations may have serious and sometimes catastrophic consequences, policy makers, educators, regulators, providers and researchers need to be aware of these factors influencing compliance. Similar to studies in other fields, such as policing, our findings support for the hypothesis that people’s law-related behavior is strongly shaped by their judgments about the legitimacy, fairness and performance of the regulatory agency [54], a proposition that was initially viewed as counterintuitive but has received widespread confirmation, initially from psychologists and more recently from a broad range of social scientists.

Based on these insights we postulate that regulatory agencies should spend further efforts in enhancing their legitimacy as it has a strong association with (self-reported) compliance behaviours. The regulatory authorities in the UAE have the opportunity to change the perceptions of their workforce and more can be done to raise awareness and improve the understanding of the role and function of the regulator. A suggested way forward is for the regulatory agencies to conduct a regular self-assessment, at least once per year, with an opportunity feedback for all participants in order to make the necessary changes and improve compliance.

Even though there is relatively limited empirical evidence which regulatory approaches work best [55], this research may assist regulatory agencies to expand their regulatory toolkit [56] and experiment with alternative ways of setting direction, monitoring compliance and enforcement. To truly measure the effects of a regulatory approach based on the procedural justice model, healthcare researchers should make use of randomized control trails to find out whether this has a meaningful impacts on perceptions and compliance. A small number of researchers [57, 58] have attempted to conduct trials in other regulatory contexts, such as policing and law. Regulatory agencies should attempt to present themselves as trustworthy and reliable actors in the healthcare field by ensuring that their directive approach is accessible and understandable, their monitoring is logical, transparent and fair, and their enforcement role is easily understood and based on evidence.

## Additional file

Additional file 1: Survey Instrument. (DOCX 24 kb)

## Abbreviation

UAE: United Arab Emirates
