# Supplementary material for: A cross-sectional study into medical students’ perceptions of healthcare regulation and self-reported compliance: a study conducted in the City of Al Ain, United Arab Emirates, 2016
Source: BMC Med Educ. 2018 Dec 13;18:305. doi: 10.1186/s12909-018-1393-x (PMC6293584; doi:10.1186/s12909-018-1393-x)
Supplement: Supplementary file 1 — Survey Instrument. (DOCX 24 kb) [file 12909_2018_1393_MOESM1_ESM.docx]

**Additional file 1 – Survey Instrument**

**Instructions**

Many international and national regulatory authorities, such as the World Health Organization (WHO), the Joint Commission International (JCIA), the Health Authority Abu Dhabi (HAAD) and the UAE Ministry of Health, attempt to govern, steer and control behaviors and activities of healthcare organizations and healthcare professionals to protect and improve the quality and safety of health care. Collectively this is also known as regulation. Regulation covers a wide range of interventions such as setting standards, targets and guidelines; conducting surveillance or assessment of the levels of compliance (through audits, inspections, etc.), and enforcing or encouraging compliance using sanctions and rewards. As a future healthcare professional, we would like to find out your views on the role regulation must play in healthcare. Please note that we are interested in your views regarding regulation in general and they do not relate to the specific context of healthcare regulation in the UAE.

This short survey will take approximately 5-10 minutes to complete. Please note we will not share the identity of those participating in the research. The information that we collect from this research project will be kept confidential.

**SECTION 1 – Your views on the regulation of healthcare**

**Please indicate your agreement or disagreement with the following statements:**

|  | **Strongly Disagree** ⯆ | | **Disagree** ⯆ | | | **Neither** ⯆ | | **Agree** ⯆ | | **Strongly Agree** ⯆ |  |
| --- | --- | --- | --- | --- | --- | --- | --- | --- | --- | --- | --- |
| 1. My friends and family would describe me as somebody who complies with rules and regulations | 🞎1 | | 🞎2 | | | 🞏3 | | 🞎4 | | 🞏5 |  |
| 2. It is hard and difficult for medical staff to comply with the rules and regulations set by regulatory authorities | 🞎1 | | 🞎2 | | | 🞏3 | | 🞎4 | | 🞏5 |  |
| 3. It is important not to let the regulatory authority push you around | 🞎1 | | 🞎2 | | | 🞏3 | | 🞎4 | | 🞏5 |  |
| 4. You should accept the decisions made by the regulatory authority, even if you think they are wrong | 🞎1 | | 🞎2 | | | 🞏3 | | 🞎4 | | 🞏5 |  |
| 5. I try very hard to follow relevant guidelines and requirements from regulatory authorities | 🞎1 | | 🞎2 | | | 🞏3 | | 🞎4 | | 🞏5 |  |
| 6. Regulations such as standards, directives and policies are needed because they have a strong, positive impact on the quality of care delivery | | 🞎1 | | 🞎2 | 🞏3 | | 🞎4 | | 🞏5 | | |
| 7. In my opinion, regulatory authorities treat all healthcare professionals and organizations fairly | | 🞎1 | | 🞎2 | 🞏3 | | 🞎4 | | 🞏5 | | |
| 8. Staff working for the regulatory authorities’ respect people's rights | | 🞎1 | | 🞎2 | 🞏3 | | 🞎4 | | 🞏5 | | |

| **SECTION 1 – Your views on the regulation of healthcare(continued)** | **Strongly Disagree** ⯆ | **Disagree** ⯆ | **Neither** ⯆ | **Agree** ⯆ | **Strongly Agree** ⯆ |
| --- | --- | --- | --- | --- | --- |
| 9. The regulatory authority should have the power to decide which regulatory requirements are the most important | 🞎1 | 🞎2 | 🞏3 | 🞎4 | 🞏5 |
| 10. In my opinion, the regulatory authorities are effective in improving the quality of health care delivery | 🞎1 | 🞎2 | 🞏3 | 🞎4 | 🞏5 |
| 11. You should always do whatever the regulator tells you to do even though you may not understand the reasons for their decisions | 🞎1 | 🞎2 | 🞏3 | 🞎4 | 🞏5 |
| 12. I normally bring concerns about safety immediately to the attention of people who are in charge | 🞎1 | 🞎2 | 🞏3 | 🞎4 | 🞏5 |
| 13. Sometimes it is OK to ignore what the regulatory authority tells you to do | 🞎1 | 🞎2 | 🞏3 | 🞎4 | 🞏5 |
| 14. The regulatory system in the UAE is not perfect, but works well | 🞎1 | 🞎2 | 🞏3 | 🞎4 | 🞏5 |
| 15. In general, I tend to comply with what is expected of me by regulatory authorities | 🞎1 | 🞎2 | 🞏3 | 🞎4 | 🞏5 |
| 16. The laws and regulation issued by the regulator are consistent (in line with) the views of residents in the UAE | 🞎1 | 🞎2 | 🞏3 | 🞎4 | 🞏5 |
| 17. Healthcare workers are unlikely to face sanctions or penalties if they do not obey the rules and regulations | 🞎1 | 🞎2 | 🞏3 | 🞎4 | 🞏5 |
| 18. It is likely that you get caught and penalized if you break any rule or regulations | 🞎1 | 🞎2 | 🞏3 | 🞎4 | 🞏5 |
| 19. The regulatory authorities in the healthcare field make their decisions based on facts, not opinions | 🞎1 | 🞎2 | 🞏3 | 🞎4 | 🞏5 |
| 20. Regulatory requirements are applied to all people consistently | 🞎1 | 🞎2 | 🞏3 | 🞎4 | 🞏5 |

**SECTION 2 – Background Information**

**This information will help in the analysis of the survey results.**

1. In the past 12 months, how often you have been in direct contact with regulatory authorities such as HAAD, DHA or the UAE Ministry of Health?

| 🞎 a. Never | 🞎 d. Weekly |
| --- | --- |
| 🞎 b. Infrequently | 🞎 e. Daily |
| 🞎 c. Monthly |  |

1. Overall, how would you rate your awareness and understanding of the current regulatory requirements in the UAE?

| 🞎 a. Very Good | 🞎 d. Fair |
| --- | --- |
| 🞎 b. Good | 🞎 e. Poor |
| 🞎 c. Average |  |

1. I would rate my own clinical skills and competencies as:

| 🞎 a. Very Good | 🞎 d. Fair |
| --- | --- |
| 🞎 b. Good | 🞎 e. Poor |
| 🞎 c. Average |  |
